# Anti-RANKL treatment inhibits erosive joint destruction and lowers inflammation but has no effect on bone formation in the delayed-type hypersensitivity arthritis (DTHA) model

**DOI:** 10.1186/s13075-016-0931-3

**Published:** 2016-01-23

**Authors:** Sara Marie Atkinson, Janine Bleil, René Maier, Anja A. Kühl, Mette Thorn, Kyle Serikawa, Brian Fox, Kim Kruse, Claus Haase, Søren Skov, Anneline Nansen, Uta Syrbe

**Affiliations:** Global Research, Novo Nordisk A/S, 2760 Måløv, Denmark; Veterinary Disease Biology, Faculty of Health and Medical Sciences, University of Copenhagen, 1870 Frederiksberg, C, Denmark; Medizinische Klinik für Gastroenterologie, Infektiologie und Rheumatologie, Charité, Campus Benjamin Franklin, Hindenburgdamm 30, 12200 Berlin, Germany; German Rheumatism Research Center, Berlin, Germany; Bioneer, Hørsholm, Denmark; Benaroya Research Institute, Seattle, WA USA; Immunexpress, Seattle, WA USA; Global Research, Novo Nordisk, Seattle, WA USA; Department of Pharmacology, Zealand Pharma, Glostrup, Denmark

**Keywords:** Arthritis, Joint inflammation, Bone destruction, Bone formation, Osteoclast, RANKL

## Abstract

**Background:**

The aims of the present study were to determine the relationship between bone destruction and bone formation in the delayed-type hypersensitivity arthritis (DTHA) model and to evaluate the effect of receptor activator of nuclear factor κB ligand (RANKL) blockade on severity of arthritis, bone destruction, and bone formation.

**Methods:**

DTHA was induced in C57BL/6 mice. Inflammation, erosive joint damage, and new bone formation were semiquantitatively scored by histology. Osteoclast activity was assessed in vivo, and messenger RNA (mRNA) expression of mediators of bone destruction and bone formation were analyzed by mRNA deep sequencing. Serum concentrations of tartrate-resistant acid phosphatase 5b, carboxy-terminal telopeptide I (CTX-I), matrix metalloproteinase 3 (MMP3), and serum amyloid P component (SAP) were determined by enzyme-linked immunosorbent assay. Anti-RANKL monoclonal antibody treatment was initiated at the time of immunization.

**Results:**

Bone destruction (MMP3 serum levels, cathepsin B activity, and RANKL mRNA) peaked at day 3 after arthritis induction, followed by a peak in cartilage destruction and bone erosion on day 5 after arthritis induction. Periarticular bone formation was observed from day 10. Induction of new bone formation indicated by enhanced Runx2, collagen X, osteocalcin, MMP2, MMP9, and MMP13 mRNA expression was observed only between days 8 and 11. Anti-RANKL treatment resulted in a modest reduction in paw and ankle swelling and a reduction of serum levels of SAP, MMP3, and CTX-I. Destruction of the subchondral bone was significantly reduced, while no effect on bone formation was seen.

**Conclusions:**

Anti-RANKL treatment prevents joint destruction but does not prevent new bone formation in the DTHA model. Thus, although occurring sequentially during the course of DTHA, bone destruction and bone formation are apparently not linked in this model.

## Background

Chronic joint inflammation in rheumatoid arthritis (RA) and spondyloarthritis (SpA) leads to disease-specific reactions in the adjacent joint structures. In RA, which mainly affects peripheral joints, chronic inflammation is characterized by synovial hyperplasia, pannus formation, cartilage destruction, and bone erosion, leading to destruction of joint architecture and function [1, 2]. In axial SpA, chronic inflammation predominantly affects the sacroiliac joints and vertebral bodies [3]. This is accompanied by local bone erosion and more prominent new bone formation, leading to joint ankylosis and syndesmophyte development [4]. In addition, new bone formation may develop at sites of peripheral enthesial inflammation in SpA [5], which is a particularly prominent finding in psoriatic arthritis [6].

Matrix-degrading enzymes, osteoclasts, and invasion of chronically activated synoviocytes are implicated in bone destruction in RA [7–9], but little is known about the mechanisms involved in inflammation-driven new bone formation, occurring mostly in SpA rather than in RA. Histological analysis of facet joints from patients with ankylosing spondylitis (AS), the progressed form of axial SpA, showed erosive damage of the subchondral bone from the bone marrow side. This damage is driven by osteoclasts located at the leading edge of a subchondral fibroblast-rich granulation tissue [10], which invades the subchondral bone. New bone formation has been linked to this subchondral pannus [11, 12], suggesting that bone destruction and new bone formation could be linked. In contrast, in mouse models of SpA, specifically in the DBA/1 model, where grouped caging of male DBA/1 mice leads to development of ankylosing enthesitis [13], destruction and bone formation do not seem to be linked, because inhibition of osteoclast function does not prevent new bone formation [14].

The delayed-type hypersensitivity arthritis (DTHA) model is a new inflammatory arthritis model in C57BL/6 mice. It combines collagen type II antibody (anti-CII) administration with local DTH induction in the footpad, which leads to severe arthritis and periarticular inflammation, including enthesial sites [15]. Arthritis development is dependent on CD4^+^ cells and proinflammatory cytokines and is characterized by bone and cartilage destruction, pannus formation, and osteophyte development at sites of periarticular inflammation. Prophylactic tumor necrosis factor α (TNFα) blockade not only reduces inflammation and bone erosion but also prevents new bone formation [15], indicating that both bone destruction and bone formation are triggered by inflammation in this model.

In this study, we determined the relationship between bone destruction and new bone formation in the DTHA model. We investigated the spatiotemporal occurrence of bone destruction and new bone formation by histological analysis, by performing an expression analysis of genes involved in tissue destruction and bone formation, and by analysis of the effect of preventive receptor activator of nuclear factor κB ligand (RANKL) blockade. Cartilage destruction and bone erosion peaked early after arthritis induction, while periarticular bone formation occurred later in the disease course. Bone erosion was accompanied by osteoclastogenesis and high levels of local bone resorptive activity, followed by induction of genes related to endochondral and direct bone formation. Anti-RANKL treatment had a modest effect on inflammation and prevented bone resorption but did not affect bone formation, indicating that bone formation and destruction are not linked in the DTHA model.

## Methods

### Animals

Female 8–10-week-old C57BL/6 J mice were purchased from Taconic (Ry, Denmark). Animals were housed in a temperature- and humidity-controlled facility with a 12-h light/dark cycle and with free access to water and standard rodent chow (Altromin®; Altromin Spezialfutter, Lage, Germany). All animal experiments were conducted according to Danish legislation and were approved by the Danish Animal Inspectorate and the Novo Nordisk ethical review board.

### Induction and assessment of DTHA

DTHA was induced as described previously [15]. In brief, mice were immunized intradermally with methylated bovine serum albumin (mBSA; Sigma-Aldrich, St. Louis, MO, USA) emulsified in Difco complete Freund’s adjuvant (CFA; BD Diagnostics, Sparks, MD, USA) at the base of the tail. Four days later, mice were given 1000 μg (approximately 50 mg/kg) of anti-mouse anti-CII cocktail (Chondrex, Redmond, WA, USA) intravenously (i.v.) in 200 μl of phosphate-buffered saline (PBS). Seven days after immunization, the mice were challenged with 200 μg of mBSA subcutaneously in 20 μl of PBS in the right footpad and as a control with 20 μl of PBS in the left footpad. Paw and ankle swelling was measured using a dial thickness gauge (Mitutoyo, Kanagawa, Japan) and change in swelling on day *x* was calculated by subtracting swelling measured on day 0 from swelling measured on day *x*.

### Blockade of RANKL

Five hundred micrograms of anti-RANKL monoclonal antibody (mAb; clone IK22/5) or rat immunoglobulin G2a (IgG2a) isotype control (Bio X Cell, West Lebanon, NH, USA) were injected intraperitoneally in 200 μl of PBS three times weekly from the time of immunization. The experimenter was blinded to the treatment groups.

### Histopathology

For histopathological evaluation, paws were fixed in 4 % paraformaldehyde, decalcified for 7 days, dehydrated, and embedded in paraffin. Sections of 3–5 μm were cut and deparaffinized for histopathological evaluation.

For overview, paws were stained with hematoxylin and eosin (H&E). For detection of cartilage destruction, Safranin O staining was performed. Collagen type X (COLX)-positive hypertrophic chondrocytes and osteocalcin-positive osteoblasts were detected by immunohistochemical staining. Osteoclasts were histochemically stained for tartrate-resistant acid phosphatase (TRAP; Kamiya Biomedical, Seattle, WA, USA). For osteocalcin staining, heat-induced epitope retrieval at 100 °C for 30 minutes in citrate buffer (pH 6.0) and subsequent enzymatic retrieval with proteinase K (Dako, Glostrup, Denmark) at 37 °C for 7 minutes was used. For COLX detection, a double enzymatic retrieval with protease (Sigma-Aldrich) in Tris-buffered saline (TBS; 2 mg/ml) at 37 °C for 15 minutes and hyaluronidase (Sigma-Aldrich) in TBS (2 mg/ml) at 37 °C for 150 minutes was used. After blocking of nonspecific binding by goat serum (10 %; Dianova, Hamburg, Germany) for 15 minutes, sections were incubated with anti-osteocalcin (polyclonal rabbit, dilution 1:500; Enzo Life Sciences AG, Lausen, Switzerland) and anti-COLX (clone X53, dilution 1:25; Quartett Immunodiagnostics, Berlin, Germany) overnight at 4 °C. After blocking of endogenous biotin with an Invitrogen avidin–biotin-blocking kit (Thermo Fisher Scientific, Paisley, UK) the slides were incubated for 1 h with species-specific biotinylated immunoglobulin (Dianova) and for 1 h with alkaline phosphatase (ALP)-streptavidin (Vector Laboratories, Burlingame, CA, USA). ALP was visualized using Chromogen Red (Dako REAL Detection System Kit; Dako) before counterstaining with Mayer’s hematoxylin. As negative controls, experiments were performed (1) with isotype controls for IgG and (2) by omitting the primary antibodies.

### Histopathological scoring of DTHA paws

Pathological changes in the metatarsal and tarsal joints of the paws were semiquantitatively assessed. The extent of inflammation and of periarticular and subchondral pannus tissue was assessed on H&E-stained sections, the extent of cartilage and bone damage was assessed on Safranin O/Fast Green–stained sections, and the extent and type of new bone formation were assessed on COLX- and osteocalcin-stained sections, each on a scale of 0–3 (no, minimal, moderate, or severe). Results are given as mean ± standard error of the mean (SEM).

### Microscope, camera, and software

Scoring was performed using an Olympus BX60 microscope (Olympus, Hamburg, Germany). Pictures were taken with a digital camera (Color View II Soft Imaging System; Olympus Soft Imaging Solutions GmbH, Münster, Germany) and analyzed using analytical software (Soft Imaging Software Cell D; Olympus Soft Imaging Solutions GmbH).

### Enzyme-linked immunosorbent assays

Serum levels of the following markers were determined employing commercially available enzyme-linked immunosorbent assays: RANKL, matrix metalloproteinase 3 (MMP3) (both from R&D Systems, Minneapolis, MN, USA), TRAP5b and carboxy terminal telopeptide I (CTX-I) [both from Immunodiagnostic Systems (IDS), Tyne & Weir, UK] and serum amyloid P component (SAP; GenWay Biotech, San Diego, CA, USA). Results are displayed as mean ± SEM.

### In vivo imaging of bone erosion activity using fluorescence molecular tomography

Mice were injected i.v. with 4 nmol of the probe Cat B 750 FAST dissolved in PBS (PerkinElmer, Waltham, MA, USA). Cat B 750 FAST is a cathepsin B activatable fluorescence agent. It is optically silent upon injection but cleaves upon contact with cathepsin B, thereby emitting a fluorescent signal. Mice were scanned approximately 20 h after probe injection. Mice were anesthetized with isoflurane and gently placed in a dorsal position in the fluorescence molecular tomography (FMT) cassette. Their paws were placed on FMT scanner blocks and kept in place with surgical tape. The cassette was inserted into the FMT scanner in a heated environment and with the mice under continuous inhalation anesthesia. The mice were scanned at 750 nm with the FMT 2000 scanner (PerkinElmer). The data are presented as the relative picomolar fluorescence in the right hind paw with the control value obtained from the left (control) hind paw subtracted.

### mRNA deep sequencing

Paws were homogenized in Qiagen Buffer RLT (Qiagen, Germantown, MD, USA) with 1 % β-mercaptoethanol (Sigma-Aldrich) and stored at −80 °C before total RNA isolation using the Ambion MagMAX preparation protocol (Thermo Fisher Scientific). After RNA quality control using an Agilent Bioanalyzer (Agilent Technologies, Santa Clara, CA, USA) and assessment of RNA quantity using a NanoDrop instrument (NanoDrop Products, Wilmington, DE, USA), RNAseq libraries were prepared from 120 ng of total RNA with an Illumina TruSeq Sample Prep Kit (Illumina, San Diego, CA, USA). Barcode adapters were added to samples in such a way as to allow pooling of samples in flow cell lanes in a randomized pattern relative to sample annotation and source. Samples were sequenced using an Illumina HiSeq 2000 system (Illumina) at a multiplexing level sufficient to generation 10 million to 25 million reads per sample. Following generation of sequences, reads were aligned to mouse genome NCBI Build 37 (mm9) using TopHat (http://tophat.cbcb.umd.edu/). Quality control of the sequencing data was accomplished by using the ShortRead package in R (http://www.r-project.org/). Aligned reads were mapped to Ensembl transcript models and converted to reads per kilobase per million values using Cufflinks (http://cufflinks.cbcb.umd.edu/).

### Statistical analysis

Statistical analyses were performed using GraphPad Prism 5 for Windows Version 5.01 software (GraphPad Software, La Jolla, CA, USA). Nonparametric data or nonnormal parametric data of two groups were analyzed by using the Mann–Whitney *U* test, and parametric data were analyzed by using a two-sided unpaired *t* test or one-way analysis of variance. For multiple group comparisons, the Kruskal–Wallis test and Dunn’s multiple comparisons test as a posttest were used. A *p* value less than 0.05 was considered significant, and levels of significance were assigned as *p ≤ 0.05, **p ≤ 0.01, and ***p ≤ 0.001.

## Results

### Pathology in DTHA is characterized by early development of periarticular and subchondral pannus tissue

To examine tissue pathology in the DTHA model, we induced DTHA as described previously [15] and performed an extensive histopathological analysis with the purpose of assessing inflammation, pannus formation, tissue destruction, and new bone formation. In brief, C57BL/6 J mice were immunized with mBSA in CFA, followed 4 days later by intravenous administration of a cocktail of anti-CII mAbs. One week after immunization, the mice were challenged with mBSA in the right hind paw and with PBS in the left hind paw as a control.

**Fig. 1:**
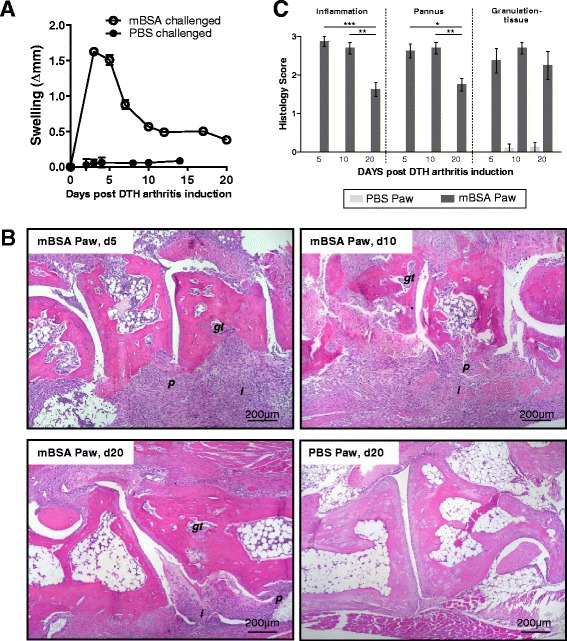
Inflammation in delayed-type hypersensitivity arthritis (DTHA) is accompanied by periarticular pannus and subchondral granulation tissue formation. DTHA was induced by intradermal immunization with methylated bovine serum albumin (mBSA) in complete Freund’s adjuvant at the tail base on day −7, followed by intravenous administration of a cocktail of collagen II antibodies on day −4 and footpad challenge with mBSA in phosphate-buffered saline (PBS; right paw) or PBS (left paw) only on day 0. **a** Paw swelling of the mBSA-challenged right hind paw and the PBS-challenged left paw at indicated time points after DTHA induction (mean ± standard error of the mean, *n* = 6–16). **b** Representative examples of hematoxylin and eosin (H&E)-stained tissue sections (original magnification, ×50) of the mBSA-challenged paw (days 5, 10, and 20) and the PBS-challenged paw (day 20). *p* periarticular pannus, *i* inflammation, *gt* subchondral granulation tissue. **c** Histopathological scoring of inflammation, extraarticular pannus, and subchondral granulation tissue at indicated time points after arthritis induction by evaluation of H&E-stained tissue sections.

A maximum of swelling of the mBSA-challenged right hind paw was observed on days 3–5 after arthritis induction (Fig. 1a). Histological assessment revealed massive synovial and extraarticular inflammation within the mBSA-challenged paw (Fig. 1b and c). Scoring of inflammation (the magnitude of the cellular infiltrate, score range 0–3) showed peak inflammation on days 5 and 10 and a decline on day 20 (Fig. 1c). Development of an inflammatory periarticular pannus tissue (i.e., a fibroblast-rich tissue containing mononuclear immune cells at synovial and periarticular sites, including enthesial attachments at apophyses) was most prominent on day 5 and day 10 and also declined by day 20 after arthritis induction (Fig. 1c). Furthermore, we noticed that the normal bone marrow had been replaced by a fibroblast-rich granulation tissue infiltrated with mononuclear cells (similar to pannus tissue at periarticular sites) in subchondral bone marrow regions adjacent to inflamed joints (Fig. 1b and c). This subchondral granulation tissue was detectable already on day 5 and did not change in magnitude throughout the study. The left paws showed no inflammation or periarticular or subchondral pannus tissue.

### Sequential development of joint destruction and new bone formation

**Fig. 2:**
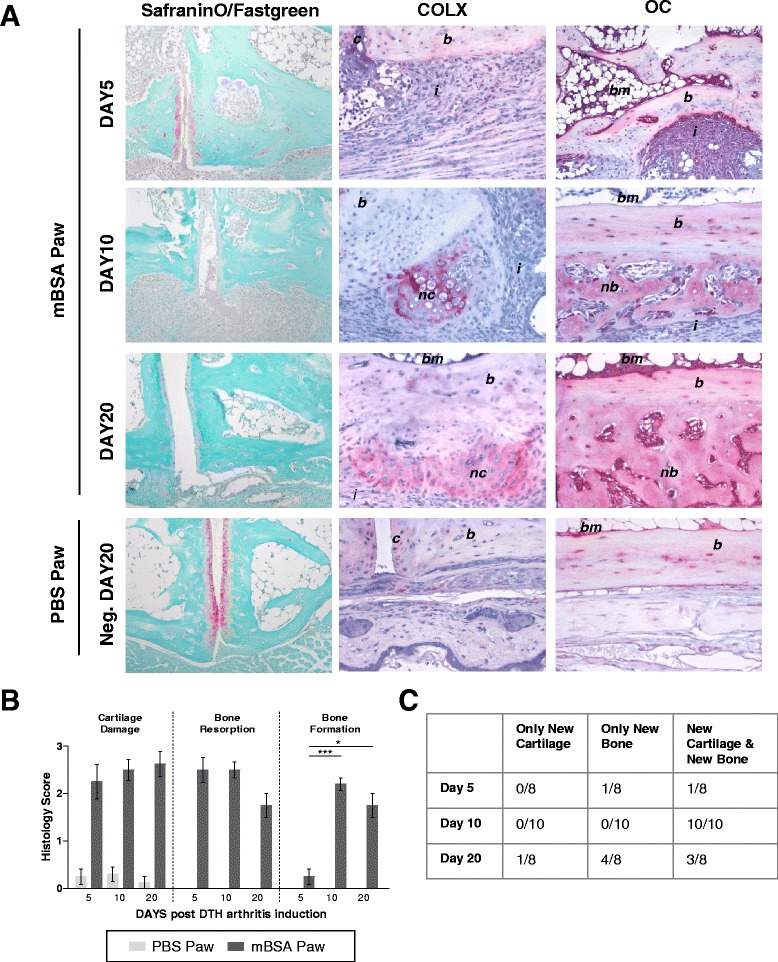
Inflammation in delayed-type hypersensitivity arthritis (DTHA) is accompanied by early cartilage damage and bone resorption that precedes new bone formation. **a** Representative examples of Safranin O/Fast Green and collagen type X (COLX)- and osteocalcin (OC)-stained tissue sections of DTHA or control [phosphate-buffered saline (PBS)] paws at indicated time points. *c* cartilage, *b* bone, *i* inflammation, *bm* bone marrow, *nc* new cartilage, *nb* new bone. Safranin O/Fast Green original magnification × 100, COLX and OC original magnification × 200. **b** Histological scores of cartilage damage and bone resorption performed on Safranin O/Fast Green–stained tissue sections and of new bone formation performed on COLX- and OC-stained sections (*n* = 10 per time point). **c** Assessment of endochondral or direct bone formation or both types in tissue sections of methylated bovine serum albumin (mBSA)-challenged paws at indicated time points assessed on COLX- and OC-stained tissue sections.

Destruction of the joint structures was assessed by looking at tissue sections stained with Safranin O/Fast Green, which stains cartilage red and bone blue. Most of the cartilage damage was seen as cartilage irregularity and loss of cartilage integrity (Fig. 2a). Bone resorption facilitated by the periarticular pannus and the subchondral granulation tissue occurred early during arthritis, as shown in the scoring of cartilage damage and bone resorption (Fig. 2b). This suggests that inflammation directly induces a strong osteoclastic and proteolytic response, which drives tissue destruction.

New bone formation was detectable from day 10 (Fig. 2a and b) and was located at diaphyseal sites and periarticularly close to apophyseal tendon insertions. New bone formation was seen as endochondral bone formation, as shown by the presence of COLX-expressing hypertrophic chondrocytes, and as direct bone formation facilitated by osteocalcin-positive osteoblasts generating new woven bone (Fig. 2a). Newly formed COLX-dependent bone was found primarily at apophyseal sites, while at diaphyseal sites newly formed woven bone with osteocalcin-positive osteoblasts was found. In the majority of joints, endochondral and direct bone formation occurred simultaneously (Fig. 2c).

In this model, new bone formation is preceded by osteoclastic and proteolytic joint destruction, and this sequence of events could suggest that bone damage and new bone formation are linked in DTHA.

### Osteoclasts located at apophyseal and subchondral sites promote bone destruction by the periarticular and subchondral pannus

**Fig. 3:**
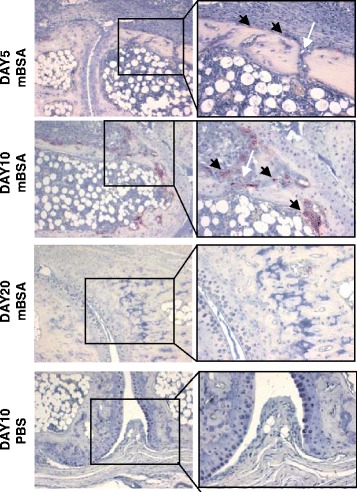
Osteoclasts are located at the edges of the periarticular and subchondral granulation tissue and at vascular channels at apophyseal sites. Tissue sections of methylated bovine serum albumin (mBSA)-challenged paws from day 5, day 10, and day 20 and of phosphate-buffered saline (PBS)-treated control paws from day 10 were stained for tartrate-resistant acid phosphatase. One representative example at × 100 original magnification (*left panels*) and × 200 original magnification (*right panels*) of indicated regions of interest is shown. *Black arrowheads* indicate osteoclasts, and *white arrow* indicates vascular channels.

Osteoclasts are involved in inflammation-driven bone destruction [16]. Activated osteoclasts, identified by TRAP expression, were detectable on day 5 and most prominently on day 10 after arthritis induction. Osteoclasts were found attached to apophyseal and diaphyseal bone adjacent to the inflamed joints at the edges of the periarticular pannus tissue and at the edges of the subchondral granulation tissue (Fig. 3). At apophyseal sites, osteoclasts were also located close to vascular channels that have been described before penetrating the bone and connecting the periarticular and/or synovial pannus with the subchondral bone marrow [17]. On day 10, activated osteoclasts were also located close to areas of direct bone formation at diaphyseal sites.

### Tissue response in DTHA involves enhanced proteolytic and osteoclastic activity and increased transcription of osteoclast- and osteoblast-specific genes

**Fig. 4:**
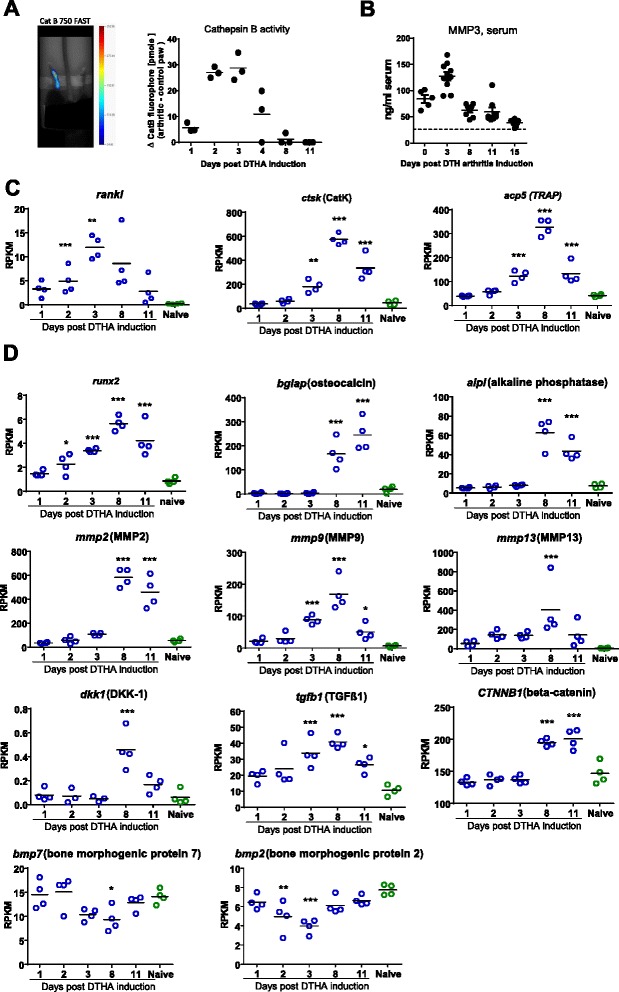
Delayed-type hypersensitivity arthritis (DTHA) is accompanied by enhanced proteolytic osteoclast activity and increased transcription of genes associated with bone erosion and new bone formation. **a** Activity of the tissue-remodeling enzyme cathepsin B (CatB; mean and individual measurements, *n* = 3–5) at indicated time points after arthritis induction in the methylated bovine serum albumin–challenged paw with subtracted fluorophore intensity of the control paw as determined by in vivo fluorescence molecular tomographic imaging. **b** Serum levels (mean ± standard error of the mean, *n* = 5–10) of matrix metalloproteinase 3 (MMP3) over the course of DTHA is shown. *Dashed line* represents the mean of naive mice (*n* = 5). **c** Messenger RNA (mRNA) expression of genes associated with bone destruction (mean and individual measurements, *n* = 4) in paw tissue as determined by mRNA deep sequencing. Significance of differences in levels in naive mice was determined using one-way analysis of variance (ANOVA) with Dunnett’s posttest for multiple comparisons. *p < 0.05; **p < 0.01; ***p < 0.001. **d** mRNA expression of genes associated with bone formation and tissue remodeling in paw tissue as determined by mRNA deep sequencing (mean and individual measurements, *n* = 4). Significance of differences in levels in naive mice was determined using one-way ANOVA with Dunnett’s posttest for multiple comparisons. *p < 0.05; **p < 0.01; ***p < 0.001. *BMP* bone morphogenetic protein; *RANKL* receptor activator of nuclear factor κB ligand, *RPKM* reads per kilobase per million reads, *TRAP* tartrate-resistant acid phosphatase, *CatK* cathepsin K, *DKK-1* dickkopf 1, *TGF-β1* transforming growth factor β1

To identify mediators involved in tissue and bone destruction, we assessed inflammatory tissue destruction by measurement of the activity of cathepsin B, a protease expressed in fibroblasts and other cells. For this purpose, we used in vivo FMT and also determined serum levels of MMP3. The peak of both local cathepsin B activity and systemic MMP3 levels was synchronized with the peak of paw swelling (Fig. 4a and b). RANKL messenger RNA (mRNA) expression in the arthritic paw followed a similar time course. In contrast, mRNA expression of TRAP and cathepsin K increased from day 3 and peaked on day 8 after arthritis induction, reflecting increased osteoclastogenesis (Fig. 4c).

Induction of osteoblast-specific genes such as ALP, osteocalcin, and β-catenin was observed only from day 8 to day 11 when transforming growth factor (TGF)-β1 mRNA expression was also increased. These events were preceded by the induction of Runx2 mRNA. Expression of *MMP2*, *MMP9*, and *MMP13* genes, which are related to the endochondral ossification pathway and bone remodeling [18–20], was also increased in the arthritic paw up to day 8 after arthritis induction. The expression of bone morphogenetic protein 2 (BMP2) and BMP7, which may act as osteoblast growth factors [21], was not increased in DTHA compared with paws of untreated naive mice, but it seemed to decrease upon arthritis induction (Fig. 4c). Interestingly, expression of the Wingless (Wnt) signaling pathway inhibitor Dickkopf (DKK)-1 was increased only on day 8, and no dysregulation was seen at early time points during acute arthritis.

### Preventive anti-RANKL treatment completely prevents bone erosion but has only a slight anti-inflammatory effect on DTHA

**Fig. 5:**
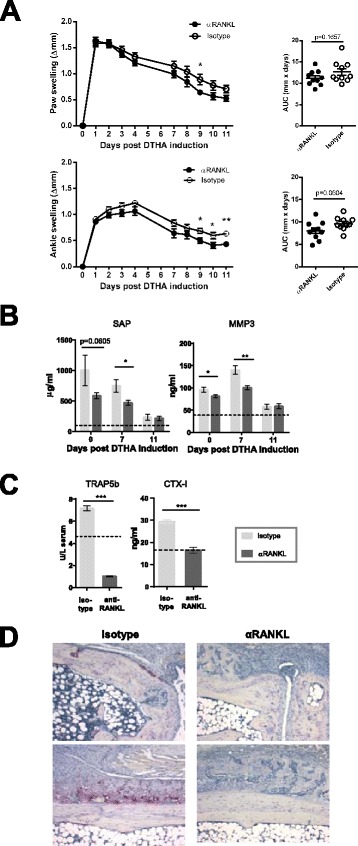
Treatment with monoclonal antibody (mAb) against receptor activator of nuclear factor κB ligand (anti-RANKL) leads to a small reduction in local and systemic inflammation and a pronounced reduction in osteoclastogenesis. Mice were treated with 500 μg of anti-RANKL mAb or isotype control in 200 μl of phosphate-buffered saline three times weekly from the time of immunization (day −7). **a** Paw (*top panel*) and ankle (*bottom panel*) swelling in mice treated with anti-RANKL or isotype control. The area under the curve (AUC) was calculated for individual mice for days 0–11 after arthritis induction, and data shown are mean ± standard error of the mean (SEM) (*n* = 10). *p < 0.05; **p < 0.01 (Student’s *t* test). **b** Serum levels (mean ± SEM, *n* = 10) of serum amyloid P component (SAP) and matrix metalloproteinase 3 (MMP3) measured on days 0, 7, and 11 after arthritis induction by enzyme-linked immunosorbent assay. *p < 0.05; **p < 0.01 (Student’s *t* test). *Dashed line* represents mean value of *n* = 10 naive mice. **c** Serum levels of tartrate-resistant acid phosphatase 5b (TRAP5b) and carboxy terminal telopeptide I (CTX-I) in serum measured on day 11 after arthritis induction. **d** Histochemical TRAP staining of paw sections from day 11 after arthritis induction from isotype and anti-RANKL-treated mice (two representative examples per treatment). Original magnification, ×100. *DTHA* delayed-type hypersensitivity arthritis

To investigate the relationship between bone destruction and new bone formation, as well as to clarify the impact of osteoclasts and specifically RANKL-dependent osteoclast activation and differentiation on arthritis severity, bone destruction, and new bone formation, we treated mice with an anti-RANKL mAb or an isotype control antibody from the time of mBSA immunization. Anti-RANKL treatment resulted in a slight reduction of paw and ankle swelling, primarily at later time points (Fig. 5a). Moreover, it also lowered levels of systemic inflammation markers compared with isotype-treated mice. This effect was more obvious at early time points, specifically at day 0 (i.e., before challenge) and at day 7 after DTHA induction (Fig. 5b). The fact that anti-RANKL treatment resulted in lower SAP and MMP3 serum levels before challenge suggests that anti-RANKL treatment also affects the response to immunization.

Treatment with anti-RANKL mAb drastically reduced TRAP5b serum levels on day 11 after arthritis induction as well as serum levels of CTX-I (Fig. 5c). Moreover, in contrast to isotype-treated mice, no TRAP-positive osteoclasts were found in arthritis paw sections from day 11 after arthritis induction (Fig. 5d).

### Preventive anti-RANKL treatment suppresses erosive joint damage and development of subchondral granulation tissue but has no effect on extraarticular new bone formation

**Fig. 6:**
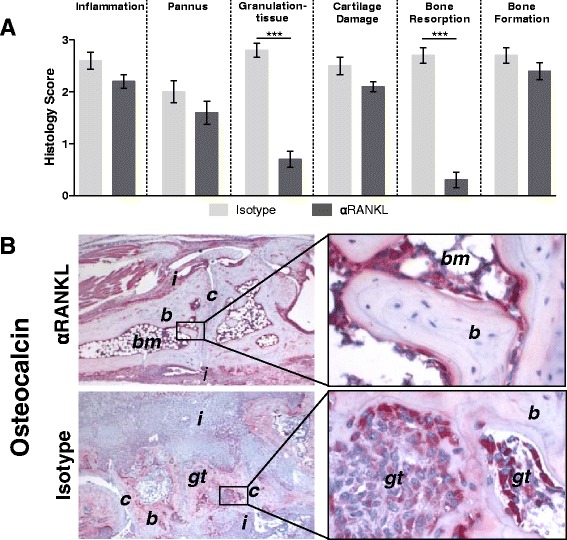
Treatment with monoclonal antibody (mAb) against receptor activator of nuclear factor κB ligand (anti-RANKL) prevents erosive joint damage in delayed-type hypersensitivity arthritis (DTHA) but has no effect on new bone formation. Mice were treated with 500 μg of anti-RANKL mAb or isotype control in 200 μl of phosphate-buffered saline three times weekly from the time of immunization (day −7). **a** Histopathological scoring of inflammation, periarticular and subchondral pannus formation, cartilage damage, bone resorption, and bone formation on day 11 after arthritis induction in paws from DTHA mice treated with either anti-RANKL or isotype control (mean ± standard error of the mean, *n* = 10). ***p < 0.001 (Student’s *t* test). **b** Representative immunohistochemical osteocalcin staining of arthritis paws on day 11 after arthritis induction in anti-RANKL- and isotype control–treated mice (*left panel* original magnification × 40, right panel original magnification × 400). *b* bone, *c* cartilage, *gt* granulation tissue, *i* inflammation, *nb* new bone

To determine the effect of anti-RANKL treatment on joint destruction and new bone formation, we scored arthritis paws from mice treated with anti-RANKL or isotype control for inflammation, pannus formation, granulation tissue development, cartilage damage, bone resorption, and bone formation. The histological scoring did not reveal any differences in inflammation between anti-RANKL- and isotype-treated mice, nor did it show differences in the extent of extraarticular pannus tissue or cartilage damage (*p* > 0.05). However, bone destruction was significantly reduced by anti-RANKL treatment (*p* < 0.001) (Fig. 6a), and a marked reduction was found in the magnitude of subchondral granulation tissue in anti-RANKL-treated mice (Fig. 6a and b). In contrast, new bone formation was completely unaffected by anti-RANKL treatment in the DTHA model, indicating that new bone formation is not linked to osteoclast activity in this model.

## Discussion

In this study, we analyzed the spatiotemporal development of joint destruction and bone formation in the DTHA model. We show that cartilage damage and bone destruction precede bone formation. Cartilage and bone damage coincide with increased local cathepsin B proteolytic activity, increased systemic MMP3 levels, and development of invasive pannus tissue periarticularly and at sites of the subchondral bone marrow. New bone development was observed at apophyseal sites mediated primarily by endochondral ossification and at diaphyseal bone sites mediated by direct bone formation. This was accompanied by upregulation of genes involved in endochondral bone formation, such as *MMP2*, *MMP9*, and *MMP13* [18–20], and by induction of osteoblast-specific genes, such as osteocalcin and ALP, in the paw tissue.

In accordance with findings in other arthritis models, we found that osteoclasts are crucially involved in joint destruction in DTHA. Osteoclast development from osteoclast precursors, monocytes, and dendritic cells (DCs) is induced by ligation of RANK by RANKL [22, 23]. RANKL was upregulated early after arthritis induction, and RANKL protein levels peaked on day 4 after arthritis induction [15]. Activated osteoclasts were seen on day 5 after arthritis induction and peaked on day 10. They were located primarily at the apophyseal attachment regions of the periarticular pannus tissue close to vascular channels penetrating the bone [17] and at the edges of the granulation tissue within the subchondral bone marrow. Treatment with anti-RANKL abrogated bone destruction in DTHA and prevented development of the subchondral granulation tissue. This suggests that osteoclast activity promotes generation of this subchondral tissue, most likely by promoting invasion of periarticular pannus tissue or inflammatory mediators into the bone marrow through the vascular channels. This seems to be a critical step in joint destruction, as the granulation tissue strongly contributes to the joint destruction process, which can be prevented by anti-RANKL treatment. Similarly to our study, anti-RANKL treatment resulted in reduced bone destruction in the collagen antibody–induced arthritis model [23] and RANKL^−/−^ mice showed reduced bone erosion in the K/BxN serum transfer arthritis model [24].

In contrast to anti-TNFα treatment, which abrogated both cartilage and bone damage [15], anti-RANKL treatment prevented only bone destruction and not cartilage damage. This suggests that cartilage damage is mediated independently of osteoclasts, most likely by proteolytic enzymes such as metalloproteinases induced by proinflammatory cytokines. However, anti-RANKL treatment also had a modest anti-inflammatory effect: It lowered systemic levels of SAP and MMP3 after immunization and mBSA challenge and resulted in slightly reduced paw swelling at later points. Although RANK–RANKL interaction may affect T effector cell generation by promoting the survival and inflammatory cytokine production of DCs [25], the fact that early paw swelling was unaffected in anti-RANKL-treated mice suggests that anti-RANKL treatment may constrain inflammation to local tissue and prevent systemic spreading rather than abrogating T-cell priming.

Inhibition of bone destruction by anti-RANKL treatment had no preventive effect on new bone formation, although bone erosion and bone formation occurred sequentially in DTHA. Similarly, blocking RANKL signaling in two different rat models of arthritis could not prevent periarticular bone formation [26], and in the DBA/1 SpA model inhibition of osteoclast activity by zoledronate did not abrogate induction of ankylosis either [14].

BMP2, which is a growth factor that stimulates chondrocyte differentiation as well as osteoblast differentiation, has been implicated in the process of bone formation and ankylosis in the DBA/1 enthesitis model. Thus, inhibition of BMP signaling by overexpression of noggin, which is an endogenous BMP antagonist, inhibited the onset and the progression of enthesitis [27]. In our study, BMP2 and BMP7 were downregulated within the arthritic paw, which is similar to findings in the collagen-induced arthritis model [28]. While BMPs were downregulated, we found upregulation of TGF-β1 after acute inflammation in DTHA had subsided. TGF-β has effects similar to those of BMPs on bone and chondrocyte precursors [29], suggesting that new bone formation may be driven by TGF-β1 in DTHA. Moreover, expression of DKK-1, a Wnt pathway inhibitor that promotes osteoblast differentiation, was not dysregulated during early arthritis but was increased at day 8, when bone formation had begun, which may indicate the presence of feedback control of the aberrant bone formation.

Thus, extraarticular new bone formation in the DTHA model is not dependent on the preceding osteoclastic damage and seems differently regulated than in the DBA/1 model of SpA. Elucidation of this mechanism is desirable, as it may provide additional clues about the development of enthesiophytes and the regulation of inflammation-driven new bone formation. The shift from inflammation to resolution of inflammation as well as the presence of extensive extraarticular tissue damage may promote new bone formation in the DTHA model. Apart from that, we provide evidence for the impact of the subchondral granulation tissue on bone destruction and the crucial role of osteoclasts for development of the subchondral bone marrow changes.

## Conclusions

Bone destruction and bone formation occur sequentially in the DTHA model. Blockade of RANKL prevents development of subchondral granulation tissue and inhibits bone destruction, but it has no effect on extraarticular new bone formation.

## Abbreviations

ALP: alkaline phosphatase
ANOVA: analysis of variance
Anti-CII: anti-collagen type II
AS: ankylosing spondylitis
AUC: area under the curve
BMP: bone morphogenetic protein
CatB: cathepsin B
CFA: complete Freund’s adjuvant
COLX: collagen type X
CTX-I: carboxy terminal telopeptide I
DC: dendritic cell
DKK-1: dickkopf 1
DTHA: delayed-type hypersensitivity arthritis
FMT: fluorescence molecular tomography
H&E: hematoxylin and eosin
IgG: immunoglobulin G
i.v.: intravenously
mAb: monoclonal antibody
mBSA: methylated bovine serum albumin
MMP: matrix metalloproteinase
mRNA: messenger RNA
OC: osteocalcin
PBS: phosphate-buffered saline
RA: rheumatoid arthritis
RANKL: receptor activator of nuclear factor κB ligand
RPKM: reads per kilobase per million reads
SAP: serum amyloid P component
SEM: standard error of the mean
SpA: spondyloarthritis
TBS: Tris-buffered saline
TGF-β: transforming growth factor β
TNFα: tumor necrosis factor α
TRAP: tartrate-resistant acid phosphatase
Wnt: Wingless
